# Muscle invasive bladder cancer in Upper Egypt: the shift in risk factors and tumor characteristics

**DOI:** 10.1186/1471-2407-8-250

**Published:** 2008-08-29

**Authors:** Ali H Zarzour, Mohie Selim, Alaa A Abd-Elsayed, Diaa A Hameed, Mohammad A AbdelAziz

**Affiliations:** 1 Public health and Community Medicine departments, Faculty of Medicine, Assiut University, Assiut, Egypt; 2 Urology, Faculty of Medicine, Assiut University, Assiut, Egypt

## Abstract

**Background:**

In Egypt, where bilharziasis is endemic, bladder cancer is the commonest cancer in males and the 2^nd ^in females; squamous cell carcinoma (SCC) is the commonest type found, with a peculiar mode of presentation. The aim of this study is to identify and rank the risk factors of muscle invasive bladder cancer (MIBC) in Upper Egypt and describe its specific criteria of presentation and histopathology.

**Methods:**

This is an analytical, hospital based, case controlled study conducted in south Egypt cancer institute through comparing MIBC cases (n = 130) with age, sex and residence matched controls (n = 260) for the presence of risk factors of MIBC. Data was collected by personal interview using a well designed questionnaire. Patients' records were reviewed for histopathology and Radiologic findings.

**Results:**

The risk factors of MIBC were positive family history [Adjusted odds ratio (AOR) = 7.7], exposure to pesticides [AOR = 6.2], bladder stones [AOR = 5], consanguinity [AOR = 3.9], recurrent cystitis [AOR = 3.1], bilharziasis [odds ratio (OR) = 5.8] and smoking [OR = 5.3]. SCC represented 67.6% of cases with burning micturition being the presenting symptom in 73.8%.

**Conclusion:**

MIBC in Upper Egypt is usually of the SCC type (although its percentage is decreasing), occurs at a younger age and presents with burning micturition rather than hematuria. Unlike the common belief, positive family history, parents' consanguinity, exposure to pesticides and chronic cystitis seem to play now more important roles than bilharziasis and smoking in the development of this disease in this area.

## Background

Bladder cancer represents a global health problem. It ranks ninth in worldwide cancer incidence. It is the 4th commonest cancer in men and the 12^th ^in women in the USA. It is estimated that about 67,160 Americans were diagnosed with bladder cancer in 2007 and 13,750 died of the disease [1].

In Egypt, carcinoma of the bladder is the most prevalent cancer, accounting for as many as 31% of all cancer cases [2]. Currently, it ranks first in males representing 16.2% of male cancer [3]. The estimated incidence in males in rural areas in Egypt is about 32 per 100.000 [4].

The exact etiology of bladder cancer is still unknown. Several risk factors have been accused as being involved in its pathogenesis such as cigarette smoking [5], synthetic nitrogen fertilizers [6], organophosphate-based pesticides [7], aromatic amines [8], pelvic irradiation, A cyclophosphamide, chronic cystitis, schistosomiasis [5], human papilloma virus [9], genetic predisposition, and some occupations [5]. The relative importance of such risk factors in the pathogenesis of the disease differs in different populations.

The aim of this study is to identify and rank the risk factors of muscle invasive bladder cancer (MIBC) in Upper Egypt and to describe the peculiarities of the disease presentation and histopathology in this specific population.

## Methods

This study is an analytical, hospital based, case control study comparing MIBC cases with matched control group in age, sex and residence for the presence of risk factors of bladder cancer.

**Figure 1:**
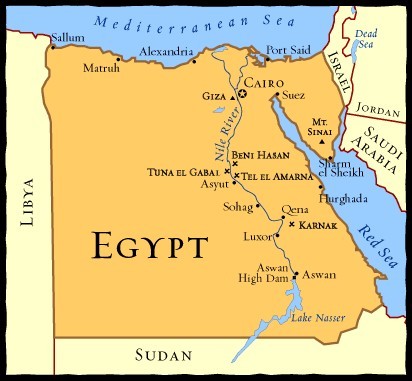
Map of Egypt.

The study was carried out in Upper Egypt which is a narrow strip of land that extends from the cataract boundaries of modern-day Aswan to the area between El-Aiyat and Zawyet Dahshur, south of modern-day Cairo, Figure 1.

The study group were residents of upper Egypt with newly diagnosed histologically proven MIBC admitted to South Egypt Cancer Institute in 2005 (n = 130).

Controls were chosen from the healthy visitors of the institute. They were matched to cases for age, sex and residence and were compared to them as regards risk factors. Two controls were matched with each case (n = 260). Full medical history, clinical examination, urinalysis and abdominal ultrasonography were done for controls to exclude the presence of any bladder lesion.

Personal interview was conducted to collect socio-demographic data (age, sex, occupation and residence), history suggestive of risk factors (bilharziasis, smoking, chronic cystitis, bladder stones, family history of cancer, parents consanguinity, exposure to chemicals, pelvic radiation and cyclophosphamide chemotherapy), and mode of presentation. History of bilharziasis is defined as finding of ovae on previous urinalysis and history of medical treatment for it. Histopathological pattern of the tumor after cystectomy was recorded.

SPSS program (version 13) was used for data analysis, which included descriptive analysis and logistic regression for calculation of risk factors.

Approval was obtained from the ethical committee of Faculty of Medicine, Assiut University. An informed written consent was obtained from all the participants, security and confidentiality of all the information obtained was guaranteed.

## Results

None of the controls had any suspicious symptom or sign of MIBC, also no suspicious lesions were found during investigating the controls.

The mean age of our patients was 58.34 ± 12.13. Males constituted 83.8% of the patients and 83.1% of controls. Residents of the rural areas were 93.8% of both patients and controls.

There was a highly significant statistical difference between cases and controls as regards the exposure to bilharziasis, fertilizer, pesticides, recurrent cystitis, bladder stones, smoking, and positive family history of bladder cancer (p < 0.001). Yet, the type of fertilizer, mode of exposure to it, the type of pesticide, mode of exposure to it, the type of smoking and the degree of relative with bladder cancer were not statistically significantly different between cases and controls.

Patients' characteristics are shown in table 1. 73.8% of cases were farmers, 86.2% were married and 91.5% were illiterates.

**Table 1:** Patients' characteristics:

Patients' Characteristics	
Occupation	
• Farmer	96 (73.8%)
• Trader	4 (3.1%)
• Employee	4(3.1%)
• House wife	22 (16.9%)
• Manual worker	4 (3.1%)

Marital status	
• Single	2 (1.5%)
• Married	112 (86.2%)
• Divorced	1 (0.8%)
• Widowed	15 (11.5%)

Education	
• Illiterate	119 (91.5%)
• Read and write	5 (3.8%)
• Primary school graduate	2 (1.6%)
• Preparatory school graduate	1 (0.8%)
• Secondary school graduate	1 (0.8%)
• Intermediate education graduate	2(1.5%)
• University graduate	0 (0%)

Risk factors of MIBC as calculated by risk estimate analysis are shown in table 2. The adjusted odds ratio (AOR) as estimated by stepwise logistic regression is shown in table 3. The most important risk factor was the positive family history of the disease (AOR 7.7, CI = 2.1–28.4, p < 0.01) followed by exposure to pesticides (AOR 6.2, CI = 3.5–11.3, p < 0.001).

**Table 2:** Risk factors of MIBC as calculated by risk estimate analysis.

	p-value	OR	(95%CI)
Positive family history of bladder cancer	p < 0.001	13.8	(4–47.7)
Exposure to pesticides	p < 0.001	9.4	(5.6–15.8)
Exposure to fertilizers	p < 0.001	7.5	(4.4–12.8)
Bladder stones	p < 0.001	7.0	(3.5–14.2)
Parents' consanguinity	p < 0.001	6.3	(3.9–10.4)
Recurrent cystitis	p < 0.001	6.1	(3.4–11.1)
Bilharziasis	p < 0.001	5.8	(3.3–10.4)
Smoking	p < 0.001	5.3	(3.2–8.7)

OR = odds ratio, CI = confidence interval.

**Table 3:** Risk factors of MIBC calculated by stepwise logistic regression:

	P-value	AOR	(95% CI)
Positive family history of bladder cancer	P < 0.01	7.7	(2.1–28.4)
Exposure to pesticides	P < 0.001	6.2	(3.5–11.3)
Bladder stones	P < 0.001	5.0	(2.2–11.4)
Parents' consanguinity	P < 0.001	3.9	(2.2–6.7)
Chronic cystitis	P < 0.01	3.1	(1.5–6.1)

AOR = adjusted odds ratio, CI = confidence interval.

The results of imaging (ultrasound, IVU and CT) and cystoscopy are shown in table 4; 77% of the patients had an obstructed kidney, 100% of them had a filling defect in IVU. CT showed single bladder lesion in 90% and multiple lesions in 10% of cases.

**Table 4:** Patients' clinical, radiological, cystoscopic, and histopathological criteria.

	(n = 130)
**First complaint**	
Burning micturition	73.8%
Haematuria	20.8%
Loin pain	3.8%
Frequency	1.6%

**Ultrasound findings**	
Visible growth	99.1
Bladder stones	8.9%
Obstructed kidney	77%

**IVU findings**	
Visible filling defect	100%
Obstructed kidney	77.3%
Normal contrast secretion	82.7%

**CT**	
Liver cirrhosisBack pressure on kidneys	20%
Back pressure on kidneys	80%
Bladder	
Single lesion	90%
Multiple lesion	10%

**Cystoscopy findings**	
Involved urethra	0%
Involved bladder neck	6.6%
Involved ureteral orifice Tumor configuration	13.1%
Solid	91.5%
Papillary	8.5%

**Pathological type**	
Well differentiated SCC	43.8%
Moderately differentiated SCC	20%
Transitional cell carcinoma	15.4%
Anaplastic carcinoma	8.5%
Spindle cell carcinoma	5.4%
Poorly differentiated SCC	3.8%
Adenocarcinoma	3.1%

**Stage of the tumor**	
T2a	8.6%
T2b	47.7%
T3a	7.8%
T3b	3.1%
T4a	28.1%
T4b	4.7%

**Lymph node affection**	
Positive	13.3%
Negative	86.7%

Table 4 also shows some important clinical and histopathological criteria of our patients. Burning micturition was the first complaint in 73.8% of cases while hematuria was the presenting symptom in only 20.8%. Digital rectal examination revealed a palpable mass in 95.4% of the cases. Squamous cell carcinoma (SCC) constituted 67.6% of cases; 47.7% of cases had a T2b tumor at the time of first presentation. Positive lymph node affection was found in 13.3% of the patients.

## Discussion

The mean age of cases in this study was 58.34 ± 12.13 years which agrees with another recent report from Egypt that found that the mean age of bladder cancer cases was 56.24 ± 11 [10]. This age is less than reported in the literature for other parts of the world; Lynch and Cohen, (1995) reported that the median ages at diagnosis for urothelial carcinoma is 69 years in males and 71 years in females [11].

The male to female ratio in this study was 5.5: 1. Residents of rural areas constituted 93.8% of cases while only 6.2% of cases lived in urban areas. This difference in male to female ratio than the international ratio of 3:1 might be explained by the fact that women in Upper Egypt aren't equally involved in farming activities with men, hence they are less exposed to the risk factors of the disease that are linked to this occupation (pesticides, fertilizers and bilharziasis) [12].

Positive family history of bladder cancer was confirmed in 13.8% of cases. Many studies reported that family history plays a major rule in developing bladder cancer and familial clusters of bladder cancer have been reported in transitional cell carcinoma (TCC) [13]. Moreover, we found that parents' consanguinity is an important risk factor for the development of bladder cancer in offspring, history of consanguinity between parents was found in 50.8% of cases (AOR = 3.9, 95%CI = 2.2–6.9, P < 0.001).

Among our patients 73.8% were farmers. In Upper Egypt, cancer risk in this occupational group is considered an important public health problem. Farmers are exposed to several hazardous substances such as fertilizers and pesticides. Moreover, the prevalence and severity of schistosomiasis tend to rise sharply with opportunities for exposure. In Egypt, the disease prevalence increased dramatically after installation of the High Dam, which created perennial irrigation instead of the basin one with subsequent higher exposure to bilharzial infestations [14]. A positive past history of bilharzial infestations was obtained from 87.7% of our cases (OR = 5.8, 95% CI = 3.3–10.4, P < 0.001). Due to the nature of the patients' work as farmers (frequently in contact with water), it is practically very difficult, if not impossible, to define the number of episodes of infestation or the time lag before each treatment course is given, also, the number of treatment courses given doesn't frequently reflect the real number of infestation episodes. Moreover, the heaviness of infestation which is believed to be an important factor in development of bladder cancer can never be practically measured. So an absolute history of exposure to bilharzial infestation was used in this study.

**Figure 2:**
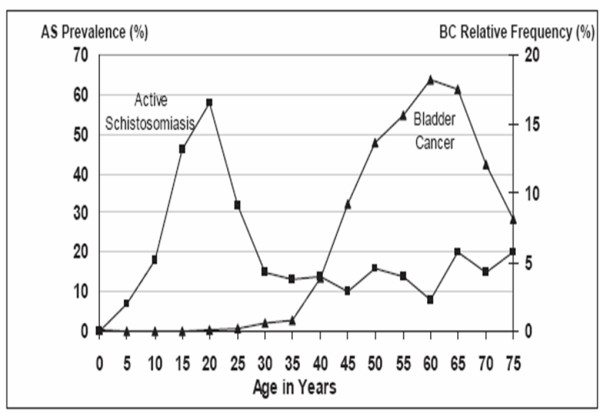
Bladder Cancer: Prevalence of Active Schistosomiasis (AS) by Age and Age Distribution of Bladder Cancer (BC) in Egypt. Ibrahim and Khaled (2006) ^17^.

There is a plethora of literature incriminating *Schistosoma haematobium *infestation as a risk factor for bladder cancer, but explanation for this association remains speculative [15]. Evidence that supports the association between schistosomiasis and bladder cancer includes the geographical correlation between the 2 conditions, the distinctive patterns of sex and age at diagnosis, the clinicopathological identity of schistosome-associated bladder cancer, and extensive evidence in experimentally infected animals [16]. The relatively high frequency of bladder cancer in Egypt supports the etiological relationship to urinary schistosomiasis. Despite the marked decrease in prevalence of endemic schistosomiasis over the last 2 decades (decreased from 35% in 1983 to 1.7% in 2003, with complete eradication in certain districts.), Egypt is still paying the toll of the previously high prevalence of the disease. Comparison of the frequency of active urinary schistosomiasis previously reported during the era of high prevalence of the disease and the age-specific incidence rate indicates a strong cohort effect, figure 2. It could be anticipated that in the near future, there will be a marked decrease in bilharziasis associated bladder cancer in Egypt as a sequel to schistosomiasis control. The potential risk is the rise in incidence of bladder cancer related to other risk factors [17].

History of exposure to pesticides was obtained in 82.3% of our patients (AOR 6.2, CI = 3.5–11.3, p < 0.001). It is well known that persons exposed to pesticides are at greater risk of developing bladder cancer than persons with no exposure to them [13].

El-Mawla et al (2001) stated that urinary bladder stones and chronic cystitis increase the risk of developing bladder cancer and particularly SCC [12]. History of bladder stones was found in 25.4% of our cases (AOR = 5, 95% CI = 2.2–11.4, P < 0.001), while 33.1% of cases had history of recurrent cystitis (AOR = 3.1, 95% CI = 1.5–6.1, P < 0.01).

Some authors have claimed that bladder carcinogenesis is related to bacterial infections, which are commonly associated with bilharzial infestation, rather than the parasite itself. Urinary bacteria have a double action: (i) the production of carcinogenic nitrosamines from their precursors in urine, e.g., nitrates and secondary amines, and (ii) the secretion of the enzyme β-glucuronidase, which may clear conjugated carcinogens, yielding free carcinogenic products [18].

Among the patients of this study, 80% were current or ex smokers (OR = 5.3, 95% CI = 3.2–8.7, P < 0.001). Cigarette smokers are reported to have up to a fourfold higher incidence of bladder cancer than do people who have never smoked [19]. Radosavljevic et al., (2003) stated that although smoking is still recognized as a major risk factor of cancers including bladder cancer, the increasing incidence of bladder cancer despite the reduction in smoking in the United States suggests that other environmental factors may be playing an increasing role in the development of bladder cancer [20]. This is in accordance with the results of this study where smoking failed to be an independent risk factor for MIBC when adjusted to other environmental factors (pesticides, fertilizers and bilharziasis).

The present data contradicts the common belief in Egypt about the major role bilharziasis plays in bladder cancer development. A larger role for the exposure to pesticides and fertilizers is evident in this study. Also the role of family history and consanguinity between parents seems to be higher than ever recognized. The risk factor profile of Egyptian bladder cancer has changed over the last 26 years as exposure to chemical carcinogens play a major role for the development of bladder cancer in Egypt [21].

Histopathological examination showed that 67.6% of cases had SCC, 15.4% TCC, 8.5% anaplastic carcinoma and 8.5% had other pathological types (table 4). This seams like a shift from what was previously reported regarding the percentage of SCC which exceeded 75% in Bilharzial bladders [22]. According to many Egyptian authors, the pattern of histopathology of bladder cancer showed a marked change over the previous years, where SCC constituted less than 60% of bladder cancer [12,21,23]. This change might be explained by the introduction of mass treatment of bilharziasis in recent years in contrast to the increased exposure to pesticides and fertilizers.

As regards the mode of presentation, globally, the most common presenting symptom of bladder cancer is painless hematuria, which occurs in about 90% of cases [24]. In this study the main presenting complaint was burning micturition, which was the presenting symptom in 73.8% of cases while hematuria was the 1st complaint in only 20.8%. This different mode of presentation might be due to the different tumor configuration which was found to be solid in 91.5% of patients. Unlike the papillary configuration which can bleed easily on shedding of the tumor cells, the solid tumor configuration delays the occurrence of hematuria. This mode of presentation complicates the picture of bladder cancer in Egypt as the patients who are usually accustomed to some kind of burning micturition due to bilharziasis, bladder stones and cystitis don't ask for medical advice until their tumors are already invasive [25]. This was reflected on the result of digital rectal examination where bladder mass was detected in 95.4% of cases.

Lymph node affection was found in only 13.3% of the patients. This might be explained by the fibrosis that affects the lymphatics in bilharzial patients. This is in agreement with the findings of other reports from Egypt [26].

## Conclusion

MIBC in Upper Egypt is peculiar in that it is usually of the SCC type (although its percentage is decreasing), occurs at a younger age and presents with burning micturition rather than hematuria.

Unlike the common belief, risk factors such as positive family history, parents' consanguinity, exposure to pesticides and chronic cystitis seem to play now more important roles than bilharziasis and smoking in the development of this disease in this area, yet reports on larger numbers of patients are needed to support this conclusion.

## Competing interests

The authors declare that they have no competing interests.

## Authors' contributions

AHZ supervised the work and gave important suggestions, MS supervised the work, participated in data analysis and manuscript writing and directed the clinical assessment, AAA-E carried out the field work, interviewing cases and controls, clinical assessment of cases and controls, data management and analysis and writing the final manuscript, DAH participated in writing the drafts, writing the final manuscript and gave very important clinical suggestions, MAA supervised the work, participated in the clinical assessment of cases and controls, directed the clinical investigations, gave very important suggestions and participated in the manuscript writing.

## Pre-publication history

The pre-publication history for this paper can be accessed here:



## References

[B1] American cancer society (2007). Cancer Facts & Figures 2007.

[B2] (1993). Public health impact of schistosomiasis: disease and mortality. WHO Expert Committee on the Control of Schistosomiasis. Bull World Health Organ.

[B3] Khaled HM (2005). Systemic management of bladder cancer in Egypt: revisited; as well as: Expert Opin Investig Drugs. J Egypt Natl Canc Inst.

[B4] Amal SI, El-Sebai I (1983). Epidemiology of bladder cancer and ligand binding.

[B5] American cancer society (2006). Detailed guide: bladder cancer. http://www.cancer.org/docroot/CRI/content/CRI_2_4_2X_What_are_the_risk_factors_for_bladder_cancer_44.asp.

[B6] Mensing TT, Speijers GJ, Meulenbelt J (2003). Health implications of exposure to environmental nitrogenous compounds. Toxicol Rev.

[B7] Webster LR, McKenzie GH, Moriarty HT (2002). Organophosphate based pesticides and genetic damage implicated in bladder cancer. Cancer Genet Cytogenet.

[B8] Wu Xifeng, Jie Lin H, Grossman Barton, Huang Maosheng, Gu Jian, Carol J, Etzel Amos CI, Dinney CP, Spitz MR (2007). Projecting Individualized Probabilities of Developing Bladder Cancer in White Individuals. Journal of Clinical Oncology.

[B9] LaRue H, Simoneau M, Fradet Y (1995). Human papilloma virus in transitional cell carcinoma of the urinary bladder. Clin Cancer Res.

[B10] El-Bolkainy MN, Akram Nouh M, El-Bolkainy T (2005). Cancer of urinary tract. Topographic Pathology of Cancer.

[B11] Lynch CF, Cohen MB (1995). Urinary system. Cancer.

[B12] El-Mawla NG, EL-Bolkainy MN, Khaled HM (2001). Bladder cancer in Africa: update. Semin Oncol.

[B13] Lynch HT, Ens JA, Lynch JF (1990). The Lynch syndrome II and urological malignancies. J Urol.

[B14] Mostafa MH, Sheweita SA, O'Connor PJ (1999). Relationship between schistosomiasis and bladder cancer. Clin Microbiol Rev.

[B15] Cheever AW (1978). Schistosomiasis and neoplasia. J Natl Cancer Inst.

[B16] Badawi AF, Mostafa MH, Probert A, O'Connor PJ (1995). Role of schistosomiasis in human bladder cancer: evidence of association, aetiological factors, and basic mechanisms of carcinogenesis. Eur J Cancer Prev.

[B17] Ibrahim AS, Khaled HM (2006). Urinary Bladder Cancer. Cancer Incidence in Four Member Countries (Cyprus, Egypt, Israel, and Jordan) of the Middle East Cancer Consortium (MECC) Compared with US SEER.

[B18] Merzabani MM, El-Aaser AA (1979). Etiological factors of bilharzial bladder cancer in Egypt: nitrosamines and their precursors in urine. Eur J Cancer.

[B19] Burch JD, Rohan TE, Howe GR, Risch HA, Hill GP, Steel R, Miller AB (1989). Risk of bladder cancer by source and type of tobacco exposure: A case-control study. Int J Cancer.

[B20] Radosavljevic V, Ilic M, Jankovic S (2003). Epidemiology and risk factors for the onset of urinary bladder cancer. Vojnosanit Pregl.

[B21] Felix AS, Soliman AS, Khaled H, Zaghloul MS, Banerjee M, El-Baradie M, El-Kalawy M, Abd-Elsayed AA, Ismail K, Hablas A, Seifeldin IA, Ramadan M, Wilson ML (2008). The changing patterns of bladder cancer in Egypt over the past 26 years. Cancer Causes Control.

[B22] El-Bolkainy MN, Mokhtar NM, Ghoneim MA, Hussein MH (1981). The impact of schistosomiasis on the pathology of bladder carcinoma. Int J Cancer.

[B23] Ghoneim MA, Abdel-Latif M, el-Mekresh M, Abol-Enein H, Mosbah A, Ashamallah A, El-Baz MA (2008). Radical cystectomy for carcinoma of the bladder: 2,720 consecutive cases 5 years later. J Urol.

[B24] Herr HW, Shipley WU, Bajorin DF (2001). Cancer of the bladder. Principles and practice of oncology.

[B25] El-Sebaie M, Zaghloul MS, Howard G, Mokhtar A (2005). Squamous cell carcinoma of the bilharzial and non-bilharzial urinary bladder: a review of etiological features, natural history, and management. Int J Clin Oncol.

[B26] Ghoneim MA, El-Makresh MM, El-Baz MA, El-Attar IA, Ashamallah A (1997). Radical cystectomy for carcinoma of the bladder. Critical evaluation of the results in 1026 cases. J Urol.

